# Analysis of risk factors of preeclampsia in pregnant women with chronic hypertension and its impact on pregnancy outcomes

**DOI:** 10.1186/s12884-024-06476-1

**Published:** 2024-04-24

**Authors:** Xiaorui Nie, Zijie Xu, Hong Ren

**Affiliations:** Department of obstetrics and gynecology Beijing Chaoyang District Maternal and Child Health Care Family Planning Service Center (Beijing Chaoyang District Maternal and Child Health Care Hospital), No. 25 Huaweili, Panjiayuan Street, Chaoyang District, Beijing, 100021 China

**Keywords:** Preeclampsia, Pregnancy, Chronic hypertension, Pregnancy outcome

## Abstract

**Objective:**

To investigate the risk factors and maternal and fetal outcomes of preeclampsia after pregnancy in patients with primary chronic hypertension.

**Methods:**

A total of 500 singleton pregnant women with a history of hypertension who were admitted for delivery at our Hospital from March 2015 to May 2022 were retrospectively collected by random sampling and divided into the non-occurrence group (*n* = 200) and the occurrence group (*n* = 300) according to whether they were complicated by preeclampsia. Afterward, the general data and the pregnancy-related data of patients were collected for comparison.

**Results:**

The univariate analysis showed significant differences between the non-occurrence group and the occurrence group in terms of the proportion of preeclampsia history (4.00% VS 24.67%, *χ2* = 37.383, *P* < 0.001), duration of hypertension > 3 years (18.00% VS 31.67%, *χ2* = 11.592, *P* < 0.001), systemic therapy (20.50% VS 10.00%, *χ2* = 10.859, *P* < 0.001), gestational age at admission [37.72 (34.10, 38.71) VS 35.01 (31.91, 37.42) weeks, Z = -9.825, *P* < 0.001]. Meanwhile, the multivariate analysis showed that a history of preeclampsia (OR = 6.796, 95% CI: 3.575 ∼ 10.134, *χ2* = 8.234, *P* < 0.001), duration of hypertension > 3 years (OR = 3.456, 95% CI: 2.157 ∼ 5.161, *χ2* = 9.348, *P* < 0.001), and a lack of systemic antihypertensive treatment (OR = 8.983, 95% CI: 7.735 ∼ 9.933, *χ2* = 9.123, *P* < 0.001) were risk factors for chronic hypertension complicated by preeclampsia during pregnancy.

**Conclusion:**

A history of preeclampsia, a longer duration of hypertension, and a lack of systematic antihypertensive treatment are risk factors for chronic hypertension complicated by preeclampsia during pregnancy. The occurrence of preeclampsia in pregnant women with chronic hypertension increases the incidence of maternal HELLP syndrome and fetal distress.

## Introduction

Chronic hypertension complicated by pregnancy refers to the occurrence of elevated blood pressure before pregnancy or before 20 weeks of gestation, with no significant aggravation during pregnancy, or diagnosed as hypertension after 20 weeks of gestation while blood pressure remains elevated for 12 weeks after delivery [1]. Studies have shown that about 6% of pregnant women in China suffer from chronic hypertension [2], and the number of patients is increasing worldwide. Meanwhile, the incidence of chronic hypertension complicated by pregnancy has increased by 67% in recent years according to foreign reports [3]. Chronic hypertension complicated by pregnancy not only affects the organ function of pregnant women but also increases the risk of adverse pregnancy outcomes. A study showed that pregnant women with a history of chronic hypertension have at least a nearly 5-fold to 6-fold increased risk of cardiovascular and cerebrovascular diseases and multi-organ dysfunction than those with normal blood pressure [4]. More studies also indicate that women with primary hypertension experience earlier and more severe maternal and fetal complications once complicated by preeclampsia after pregnancy [5]. The previous research conducted by Bramham K et al. [6] in the United States on 822 pregnant women with chronic hypertension already demonstrated that the potential risk of severe intrauterine hypoxia, fetal growth restriction, and even stillbirth was significantly increased in pregnant women with preeclampsia [7]. The incidence of preeclampsia in the general Chinese pregnant population is about 3 − 5%, while the incidence of preeclampsia in pregnant women with chronic hypertension can reach 17 − 25% [8]. In contrast, foreign studies have indicated an incidence of 13 − 40% for preeclampsia in women with chronic hypertension [9].

Preeclampsia is a complex and dynamic process, and its exact pathogenesis remains unclear. Currently, it is believed that the infiltration of trophoblast cells and the remodeling of uterine spiral arteries during placental formation are important links in the occurrence of preeclampsia [2]. Some scholars have proposed the “two-stage” theory for the occurrence of preeclampsia [10] and further developed the “six-stage” model [11]. In addition to causing adverse pregnancy outcomes, the occurrence of preeclampsia also has some impact on the long-term prognosis of pregnant patients, such as depression [12–15]. Therefore, it is of great significance to identify high-risk factors for chronic hypertension complicated by preeclampsia, screen out high-risk pregnant women promptly, and take early intervention measures for the pregnancy outcomes and long-term prognosis of both mother and child.

Most current research and clinical trials focus on statistical analysis of pregnancy-induced hypertension with or without preeclampsia and comparisons of different pregnancy outcomes. However, there is limited research on the special population of chronic hypertension with preeclampsia. Therefore, the risk factors for the occurrence of preeclampsia in pregnant women with chronic hypertension have not been clearly identified. This study is designed to analyze the population of pregnant women with chronic hypertension and explore the high-risk factors for the occurrence of preeclampsia, as well as its impact on maternal and fetal outcomes, so as to better guide the screening of high-risk groups before pregnancy and reduce the occurrence of adverse maternal and fetal outcomes.

## Study subjects and methods

### Study subjects

A total of 500 singleton pregnant women with a history of hypertension who were admitted for delivery at Beijing Chaoyang District Maternal and Child Health Care Hospital from March 2015 to May 2022 were retrospectively collected by random sampling and divided into the non-occurrence group (*n* = 200) and the occurrence group (*n* = 300) based on whether they developed concurrent preeclampsia. Inclusion Criteria: (1) Patients with a singleton pregnancy; (2) Patients diagnosed with chronic hypertension complicated by pregnancy (without preeclampsia) or chronic hypertension complicated by pregnancy with preeclampsia. Exclusion Criteria: (1) Patients with secondary hypertension such as renal hypertension and pheochromocytoma diagnosed during pregnancy or postpartum; (2) Patients with normal maternal blood pressure during follow-up at 12 weeks after delivery; (3) Patients with malformed infants found in the second trimester requiring induction of labor; (4) Patients with multiple pregnancies.

### Study methods

Diagnostic criteria for chronic hypertension: Diastolic blood pressure ≥ 90 mmHg and/or systolic blood pressure ≥ 140 mmHg before pregnancy or before 20 weeks of gestation, with the latter seeing persistent blood pressure ≥ 140/90 mmHg 3 months after delivery.

Criteria for cardiovascular risk stratification of chronic hypertension: (1) Mild (low risk): diastolic blood pressure = 90 ∼ 99 mmHg, systolic blood pressure = 140 ∼ 159 mmHg, without target organ damage; (2) Moderate (medium risk): diastolic blood pressure = 100 ∼ 109 mmHg, systolic blood pressure = 160 ∼ 179 mmHg; (3) Severe (high risk): diastolic blood pressure ≥ 110 mmHg, systolic blood pressure ≥ 180 mmHg, without target organ damage, or diastolic blood pressure = 90 ∼ 99 mmHg, systolic blood pressure = 140 ∼ 150 mmHg, with diabetes or target organ damage.

Diagnostic criteria for preeclampsia: (1) Presence of proteinuria after 20 weeks of gestation; (2) pregnant women with hypertension and proteinuria before 20 weeks of gestation suddenly developed elevated blood pressure or increased proteinuria; (3) Elevated creatinine and liver enzymes, or platelets < 100 × 109/L, or accompanied by right upper abdominal pain, headache, and other symptoms.

Treatment methods for hypertension [16]: All pregnant women were treated with low-dose aspirin (100 mg/d) before 20 weeks of gestation. For patients with mild hypertension, low-dose aspirin alone was used; for patients with blood pressure ≥ 150/100 mmHg for more than 2 times and target organ damage, antihypertensive drugs were added; for patients with a sharp increase in blood pressure or proteinuria, additional tests were conducted to determine if they had preeclampsia. Those with preeclampsia were hospitalized for observation, and if the condition stabilized, they could continue the pregnancy and be discharged with follow-up. However, they could continue hospitalization if severe preeclampsia develops. Low-dose aspirin was discontinued after 36 weeks of gestation in pregnant women without preeclampsia, with premature infants and low birth weight infants transferred to the neonatal ICU for treatment.

### Data collection

General characteristics data of the patients were collected, including: age, pre-delivery BMI, previous pregnancy and delivery history, previous blood pressure control, family history of hypertension, and history of previous preeclampsia in pregnancy. Delivery-related data included: newborn birth weight, newborn Apgar score results, and the occurrence of related maternal and fetal complications.

The newborn Apgar score is used to determine the severity of neonatal asphyxia and asphyxia, which is based on five signs within one minute after birth, i.e., heart rate, respiration, muscle tone, laryngeal reflex, and skin color, each of which scored from 0 to 2, with a maximum score of 10. Scores of 8 ∼ 10: normal; 4 ∼ 7: mild asphyxia; 0 ∼ 3: severe asphyxia. The lower the score, the more severe acidosis and hypoxemia, and the higher the mortality rate, which required immediate rescue to improve asphyxia [17].

Common maternal complications include placental abruption, postpartum hemorrhage, and HELLP syndrome, while fetal complications mainly include intrauterine growth restriction (IUGR), fetal distress, abnormal amniotic fluid volume, abnormal diastolic blood flow signals, premature delivery, and stillbirth. (1) Diagnostic criteria for placental abruption: the placental abruption surface does not exceed 1/3 of the placental area, the signs are not obvious, the main symptoms are a large amount of vaginal bleeding, no abdominal pain or mild abdominal pain, and the fetal heart rate is normal [18]; (2) Diagnostic criteria for postpartum hemorrhage: the amount of blood loss reaches or exceeds 500 mL within 24 h after delivery of the fetus [18]. (3) Diagnostic criteria for fetal growth restriction (FGR): fetal head circumference, femur length, and abdominal circumference are measured by ultrasound, and fetal weight (EFW) is estimated by substituting the HadlockC formula, and FGR is diagnosed if EFW is less than the 10th percentile of the same gestational age [19]. (4) Diagnostic criteria for fetal distress: the fetus in the womb due to acute or chronic hypoxia endangering health and life syndrome, mainly manifested as abnormal fetal heart rate during delivery, amniotic fluid meconium contamination and abnormal fetal heart rate monitoring (late deceleration, frequent mutation deceleration), abnormal fetal movement (hypoxia in the initial stage of frequent fetal movements, then weakened and reduced, and then disappeared). Intrauterine distress is diagnosed if any of the above are achieved [18]. (5) Diagnostic criteria for oligohydramnios: those with less than 300 ml of amniotic fluid in the third trimester of pregnancy are called oligohydramnios. The prenatal diagnosis of oligohydramnios is mainly determined by the maximum depth (AFV) of the dark area of the amniotic fluid and the index of amniotic fluid (AFI) by color ultrasound, with AFV ≦ 2 cm being oligohydramnios and AFI ≦ 5 cm being the standard for oligohydramnios. Hypohydramnios can be clearly diagnosed if the total amount is less than 300 ml when the membrane is broken and the amount of amniotic fluid is collected by various methods after delivery. Polyhydramnios: The amount of amniotic fluid during pregnancy is more than 2000 ml, which is called polyhydramnios. The prenatal diagnosis of polyhydramnios is mainly determined by the maximum depth (AFV) of the dark area of the amniotic fluid and the amniotic fluid index (AFI) monitored by color ultrasound, AFV ≧ 8 cm is diagnosed as polyhydramnios, and AFI ≧ 25 cm is diagnosed as polyhydramnios. A total amount of more than 2000 mL of amniotic fluid collected postpartum confirms the diagnosis of polyhydramnios [18].(6) Premature delivery: It refers to delivery at 28 weeks of gestation and less than 37 weeks of gestation, resulting in premature infants with a birth weight between 1,000 and 2,499 g [20].

### Statistical analysis

SPSS 26.00 software was used for statistical analysis. The K-S method was used for the normality test, measurement data complying with normality were expressed as (x ± s), and the independent sample t-test was used for inter-group comparison. Count data were expressed as frequency (n) or rate (%), with the *χ2* test used for the comparison of general data and the rank-sum test for the comparison of grade data. Meanwhile, the skewed distribution was described using the median (interquartile range), and the rank-sum test was used for inter-group comparison. In addition, multivariate analysis was conducted using Logistic regression, and the ROC curve was used to analyze the predictive value of each factor for the occurrence of preeclampsia in patients with chronic hypertension during pregnancy. The significance level was set at α = 0.05.

## Results

### Comparison of general data between patients with/without preeclampsia

The comparison of general data between the two groups showed that the mean age of the group without preeclampsia was 33.69 ± 5.80, with 104 primiparas, a median pre-delivery BMI of 30.00 (27.38, 32.23) kg/m2, and a history of preeclampsia (*n* = 8); the mean age of the group with preeclampsia was 32.74 ± 5.25, with 146 primiparas, the median pre-delivery BMI of 31.51 (29.21, 34.91) kg/m2, and a history of preeclampsia (*n* = 74). There were significant differences between the two groups in the history of preeclampsia (4.00% VS 24.67%, *χ*^*2*^ = 37.383, *P* < 0.001), duration of hypertension > 3 years (18.00% VS 31.67%, *χ*^*2*^ = 11.592, *P* < 0.001), systemic therapy (20.50% VS 10.00%, *χ*^*2*^ = 10.859, *P* < 0.001), and gestational age at admission (37.72 (34.10, 38.71) VS 35.01 (31.91, 37.42) weeks, Z = -9.825, *P* < 0.001). However, no significant difference was found in factors such as age, proportion of primiparous, pre-delivery BMI, proportion of positive antiphospholipid antibody, gravidity, parity, and family history of hypertension between the two groups (*P* > 0.05), as shown in Table 1.

**Table 1:** Comparison of General Data between the two Groups

Variable	Non-occurrence Group (*n* = 200)	Occurrence group (*n* = 300)	t/χ^2^/Z value	P value
Age (years-old, x ± s)	33.69 ± 5.80	32.74 ± 5.25	1.557	0.120
Primipara (n, %)	104(52.00)	146(48.70)	0.533	0.465
Pre-delivery BMI [kg/m^2^, M (Q1, Q3)]	30.00 (27.38, 32.23)	31.51 (29.21, 34.91)	-1.610	0.107
History of preeclampsia (n, %)	8(4.00)	74(24.67)	37.383	< 0.001
Positive antiphospholipid antibody (n, %)	5(2.50)	13(4.33)	1.162	0.281
Gravidity [n, M (Q1, Q3)]	2.00 (1.00, 3.00)	2.00 (1.00, 3.00)	-1.159	0.246
Parity [n, M (Q1, Q3)]	0.00 (0.00, 1.00)	0.00 (0.00, 1.00)	-0.531	0.595
Family history of hypertension (n, %)	3(1.50)	5(1.67)	0.021	0.884
Duration of hypertension > 3 years (n, %)	36(18.00)	95(31.67)	11.592	< 0.001
Systemic treatment (n, %)	41(20.50)	30(10.00)	10.859	< 0.001
Gestational age at admission [Weeks, M (Q1, Q3)]	37.72 (34.10, 38.71)	35.01 (31.91, 37.42)	-9.825	< 0.001

### Multivariate logistic regression analysis of patients with chronic hypertension complicated by preeclampsia

A logistic regression analysis model was constructed with the presence or absence of preeclampsia as the dependent variable (occurrence = 1, absence = 0), and statistically significant factors in the aforementioned analysis as independent variables [history of preeclampsia (presence = 1, absence = 0), duration of hypertension > 3 years (presence = 1, absence = 0), performing systemic therapy (presence = 1, absence = 0), and gestational age at admission entered at the original value]. The findings revealed that a history of preeclampsia (OR = 6.796, 95% CI: 3.575 ∼ 10.134, *χ*^*2*^ = 8.234, *P* < 0.001), duration of hypertension > 3 years (OR = 3.456, 95% CI: 2.157 ∼ 5.161, *χ*^*2*^ = 9.348, *P* < 0.001), and a lack of systemic antihypertensive treatment (OR = 8.983, 95% CI: 7.735 ∼ 9.933, *χ*^*2*^ = 9.123, *P* < 0.001) were risk factors for the occurrence of chronic hypertension complicated by preeclampsia. See Table 2.

**Table 2:** Multivariate Logistic Regression Analysis of Patients with Chronic Hypertension Complicated by Preeclampsia

Influencing Factors	S.E	Wald χ^2^	P value	OR	OR(95%CI)
History of preeclampsia	1.311	8.234	0.001	6.796	3.575 ∼ 10.134
Duration of hypertension > 3 years	1.213	9.348	0.001	3.456	2.157 ∼ 5.161
Lack of systemic antihypertensive treatment	0.978	9.123	0.001	8.983	7.735 ∼ 9.933
Gestational age at admission	3.978	0.123	0.924	1.283	0.435 ∼ 2.933

### Predictive value of duration of hypertension, history of preeclampsia, and systemic therapy for chronic hypertension complicated by preeclampsia in pregnant women

The duration of hypertension, history of preeclampsia, and systemic therapy were of certain value in predicting the occurrence of preeclampsia in pregnant women with chronic hypertension (*P* < 0.05). Specifically, the area under the curve (AUC) of the duration of hypertension, history of preeclampsia, and systemic therapy in predicting preeclampsia in pregnant women with chronic hypertension were 0.752 (95% CI: 0.683, 0.792), 0.746 (95% CI: 0.679, 0.821), and 0.756 (95% CI: 0.691, 0.823), respectively. In terms of combined prediction, the combined prediction effect was better than that of the single prediction, and the AUC of the three factors combined for predicting the occurrence of preeclampsia in pregnant women with chronic hypertension was 0.856 (95%CI: 0.790, 0.898), as shown in Table 3.

**Table 3:** Predictive Value of Duration of Hypertension, History of Preeclampsia, and Systemic Treatment for Chronic Hypertension Complicated by Preeclampsia in Pregnant Women

Variable	AUC	95%CI	Truncated Value	Sensitivity (%)	Specificity (%)	PPV (%)	NPV (%)
Duration of Hypertension	0.752	(0.683, 0.792)	3.64	82.43	59.53	67.07	77.21
History of Preeclampsia	0.746	(0.679, 0.821)	-	83.41	61.23	68.27	78.68
Systemic Treatment	0.756	(0.691, 0.823)	-	72.66	78.31	77.01	74.12
Combination of the Three variables	0.856	(0.790, 0.898)	-	85.42	81.42	82.13	84.81

Note: PPV: Positive predictive value; NPV: Negative predictive value

### Effect of preeclampsia on pregnancy outcomes in pregnant women with chronic hypertension

A comparison of pregnancy outcomes between pregnant women with and without preeclampsia in chronic hypertension showed that there were significant differences in the occurrence of HELLP syndrome (1 VS 22 cases, *χ*^*2*^ = 12.769, *P* < 0.001), gestational age at delivery [38.10 (36.11, 38.91) VS 35.61 (32.42, 37.61) weeks, Z = -9.874, *P* < 0.001], newborn birth weight (3200.32 ± 956.81 VS 2513.41 ± 903.82 g, t = -7.452, *P* < 0.001), 1-min newborn Apgar score (9.41 ± 0.54 VS 8.46 ± 0.33 points, t = 9.417, *P* < 0.001), 5-min newborn Apgar score (9.67 ± 0.45 VS 9.12 ± 0.24 points, t = 8.593, *P* < 0.001), umbilical artery S/D value [2.21 (1.91, 2.50) VS 2.61 (2.22, 2.93), *Z*=-9.523, *P* < 0.001], abnormal amniotic fluid volume (2 VS 15 cases, *χ*^*2*^ = 5.846, *P* = 0.016), and fetal distress (4 VS 42 cases, *χ*^*2*^ = 20.686, *P* < 0.001) were significantly different. However, there was no significant difference in postpartum hemorrhage, placental abruption, stillbirth, and IUGR between the two groups (*P* > 0.05). See Table 4.

**Table 4:** Effect of Preeclampsia on Pregnancy Outcomes in Pregnant Women with Chronic Hypertension

Index	Non-occurrence group (*n* = 200)	Occurrence group (*n* = 300)	t/χ^2^/Z value	P value
Maternal Complications				
Postpartum hemorrhage (n, %)	8(4.00)	11(3.67)	0.036	0.849
Placental abruption (n, %)	5(2.50)	13(4.33)	1.162	0.281
HELLP syndrome (n, %)	1(0.50)	22(7.33)	12.769	< 0.001
Fetal Outcomes				
Gestational age at delivery [weeks, M (Q1, Q3)]	38.10 (36.11, 38.91)	35.61 (32.42, 37.61)	-9.874	< 0.001
Newborn birth weight (g, x ± s)	3200.32 ± 956.81	2513.41 ± 903.82	-7.452	< 0.001
1-min newborn Apgar score (points, x ± s)	9.41 ± 0.54	8.46 ± 0.33	9.417	< 0.001
5-min newborn Apgar score (points, x ± s)	9.67 ± 0.45	9.12 ± 0.24	8.593	< 0.001
Intrauterine Conditions				
Umbilical artery S/D value [M (Q1, Q3)]	2.21 (1.91, 2.50)	2.61 (2.22, 2.93)	-9.523	< 0.001
Abnormal amniotic fluid volume (n, %)	2(1.00)	15(5.00)	5.846	0.016
Fetal distress (n, %)	4(2.00)	42(14.00)	20.686	< 0.001
Stillbirth (n, %)	3(1.50)	9(3.00)	1.153	0.283
IUGR (n, %)	6(3.00)	20(6.67)	3.273	0.070

### Discussion

Chronic hypertension is a lifelong disease that affects the function of various organs in the body and is the leading disease that endangers people’s health, it has been reported in literature that the incidence of adverse pregnancy outcomes increases with the elevated blood pressure levels in pregnant women with different degrees of hypertension [21]. The relationship between hypertension and preeclampsia (PE) is traceable. In 2017, Boriboonhirunsarn et al. [22] performed a statistical analysis on 300 singleton pregnant women diagnosed with primary hypertension, which showed an incidence of 43.3% for concurrent PE in pregnant women with primary hypertension, and the incidence of adverse fetal outcomes increased. Numerous reports have shown that pregnant women complicated by PE see a significantly increased risk of maternal complications such as placental abruption and fetal growth restriction, leading to poor perinatal outcomes like premature delivery [23], cardiovascular disease [24]. Therefore, monitoring and management of pregnant women with chronic hypertension complicated by preeclampsia has become a common concern worldwide.

Some studies have found [25] that the aggregation of risk factors greatly increases the occurrence of adverse maternal and fetal outcomes in preeclampsia. Therefore, identifying high-risk factors for preeclampsia and strengthening prenatal monitoring of high-risk patients have become a hot topic in obstetric research. In our study, we identified several risk factors according to analyses of univariate cox regression and multiple cox regression, among them, the discovery on the risky nature of PE history aligned with previous established researches [26, 27]. Though the negative effects of hypertension duration and patient age have been proved in PE development among hypertension patients [28], there is still a gap in related researches based on chronic hypertension population. We successfully illustrated the promoting effect of hypertension duration (> 3 years) to PE occurrence on chronic hypertension subjects, while the patient age showed no significant contribution, according to the investigation outcomes of this study. Moreover, for pregnant women with mild to moderate primary hypertension, strict and reasonable control of blood pressure levels can also reduce the incidence of preeclampsia [29], it seems to be equally applicable to pregnant subjects with chronic hypertension. The findings of this study demonstrated that untreated chronic hypertension and a longer course of hypertension increase the incidence of preeclampsia in patients with chronic hypertension.

In addition, it has been shown that family history of preeclampsia, history of preeclampsia, pregnancy interval ≥ 10 years, and primiparity are all risk factors for developing preeclampsia [29]. This study also found that a history of preeclampsia is a risk factor for developing preeclampsia after pregnancy in patients with primary chronic hypertension, which is consistent with previous research findings.

During a normal pregnancy, umbilical artery blood flow resistance gradually decreases with increasing gestational age, reflecting an increase in maternal-fetal blood exchange and normal fetal growth and development [30]. Studies have shown that the umbilical artery resistance index (S/D) should be ≤ 3 after 30 weeks of gestation [31]. This index is also a major parameter reflecting the fetal-placental circulation status and plays a role in predicting pregnancy outcomes. If this value shows no downward trend or even increases in the third trimester of pregnancy, it indicates increased blood flow resistance and increased placental perfusion pressure. This may manifest as reversed or absent umbilical artery blood flow signals, and in severe cases, it can lead to fetal hypoxia and distress.

In patients with chronic hypertension after pregnancy, due to long-term maintenance of high blood pressure levels, the small blood vessels throughout the body are in a state of spasm, affecting multiple organs and causing corresponding changes in their functions. Once complicated by preeclampsia, maternal and fetal complications (e.g., placental abruption, postpartum hemorrhage, HELLP syndrome) tend to occur earlier and more severely [32]. In order to balance the risks between the mother and the perinatal outcomes, they often lead to preterm termination of pregnancy, resulting in an increase in preterm delivery and cesarean section. The findings of this study also revealed that pregnant women with chronic hypertension complicated by preeclampsia experience a higher proportion of HELLP syndrome and shorter gestational age at delivery, which is consistent with the above findings.

Chronic hypertension complicated with preeclampsia is a common cause of iatrogenic preterm delivery, and the gestational age directly affects the maturity of the fetus, which is crucial for perinatal outcomes. Many studies have reported that preeclampsia increases the risk of fetal complications (e.g., fetal growth restriction, fetal distress, stillbirth, etc.). A statistical analysis of clinical mortality in China comparing pre-eclampsia with or without chronic hypertension suggested [33] that the probability of neonatal death due to chronic hypertension complicated with preeclampsia may be the highest among the studies of the classification of hypertensive disorders of pregnancy at various stages. Some scholars believe that chronic hypertension itself may not necessarily increase the incidence of IUGR. Only when preeclampsia causes insufficient early placental blood flow in the mother, it may become a key factor in the occurrence of fetal growth restriction [34]. The findings of of this study revealed that newborn birth weight and Apgar scores were relatively lower and the incidence of fetal distress events was higher in patients with chronic hypertension complicated by preeclampsia. However, there was no significant increase in the risk of complications such as severe fetal growth restriction and stillbirth.

Of course, this study comes with some limitations. Firstly, it is a single-center retrospective analysis, relying on medical records and laboratory query systems for data collection, and there was missing data for some pregnant women. Secondly, since its sample size is relatively small, further large-scale studies are needed to increase the persuasiveness. Specifically, further multi-center large-scale prospective studies are needed in the future.

In conclusion, a history of pre-eclampsia, long duration of hypertension, and a lack of systematic antihypertensive treatment are risk factors for chronic hypertension complicated with pre-eclampsia during pregnancy. The occurrence of preeclampsia in pregnant women with chronic hypertension increases the incidence of maternal HELLP syndrome and fetal distress.

## Data Availability

All data generated or analyzed during this study are included in this article.
